# Inflammatory myofibroblastic tumor of the lacrimal gland: case report of an exceptional location

**DOI:** 10.1186/s12907-017-0050-3

**Published:** 2017-08-17

**Authors:** Adil Boudhas, Mohamed Allaoui, Fouad El Asri, Issam Rharrassi, Mohamed Reda El Ochi, Mohamed Tbouda, Hafsa Chahdi, Abderrahmane Al Bouzidi, Mohamed Oukabli

**Affiliations:** 1 Department of Pathology, Military General Hospital Mohammed V, Mohammed V- Souissi University, Hay Riad, Postal code 10000 Rabat, Morocco; 2 Department of Ophthalmology, Military General Hospital Mohammed V, Mohammed V- Souissi University, Rabat, Morocco

**Keywords:** Inflammatory myofibroblastic tumour, Lacrimal gland, Orbit, ALK

## Abstract

**Background:**

Inflammatory myofibroblastic tumour (IMT) is a mesenchymal neoplasm of intermediate biological potential that may affect a wide range of anatomic sites but has a particular predilection for the lung and intra-abdominal soft tissues.

**Case presentation:**

We report here an exceptional case of inflammatory myofibroblastic tumor arising in the lacrimal gland and presenting as an orbital mass in a 24-year-old male.

**Conclusion:**

This report aims to discuss the importance of histopathological and immunohistochemical findings in arriving at the diagnosis, which helps dictate the management, treatment and prognosis of the patient.

## Background

Inflammatory myofibroblastic tumor (IMT) is a very rare mesenchymal tumour that can arise in various anatomic locations [1, 2]. IMT is characterized by a proliferation of myofibroblastic cells admixed with inflammatory elements. Approximately half of IMTs have a rearrangement of the anaplastic lymphoma kinase (ALK). Moreover, this entity is quite curable if completely and appropriately excised [1–3]. We present here an exceptional case of orbital IMT occuring in the lacrimal gland.

## Case presentation

A 24-year-old male, was referred to our medical institution with a 4 months history of progressive and indolent mass in the super-external area of the left eye with no notion of trauma or insect bite.

**Fig. 1:**
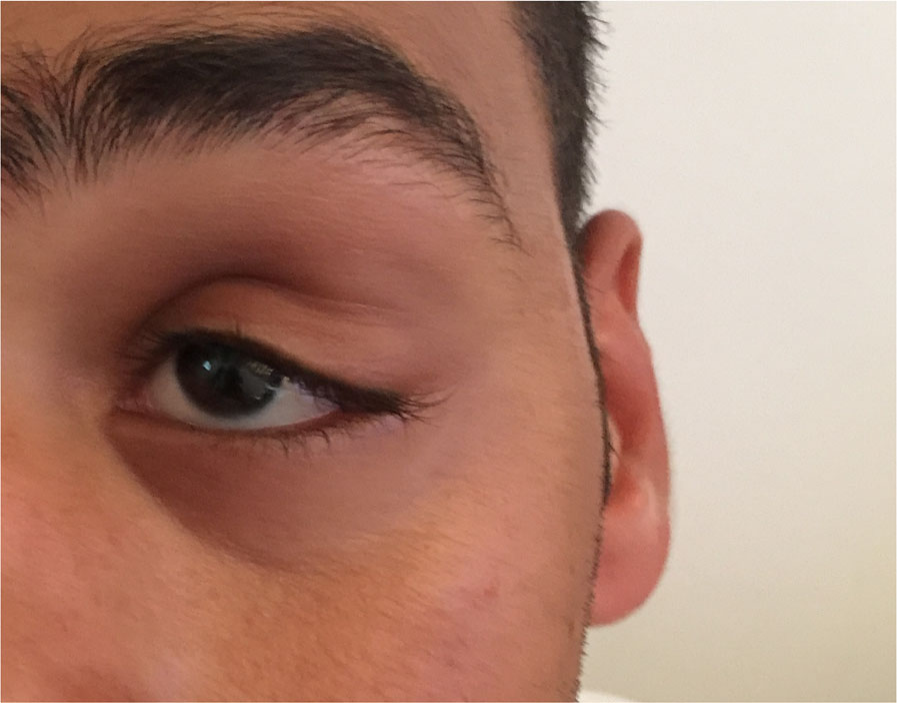
Picture showing the patient with swelling at the superolateral angle of the left orbit

On clinical examination, there was a soft tissue swelling at the superolateral angle of the left orbit (Fig. 1). The patient also had left upper eyelid ptosis with no associated diplopia, proptosis or other clinical symptoms. Pupils were round and reactive. Visual acuity was 20/20 in each eye. Visual fields and intraocular pressures were normal.

**Fig. 2:**
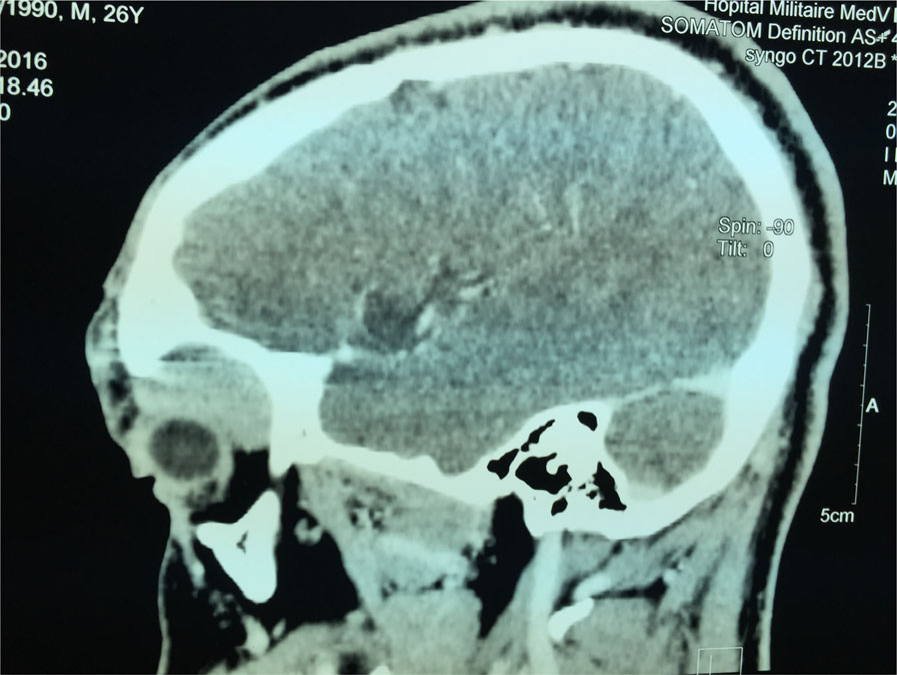
Computed Tomography scan showing a well circumscribed and homogeneous left supraconal tumor

Computerized tomography (CT) scan revealed a well circumscribed and homogeneous left lacrimal gland tumor without bone erosion, measuring 22 mm × 21 mm (Fig. 2).

**Fig. 3:**
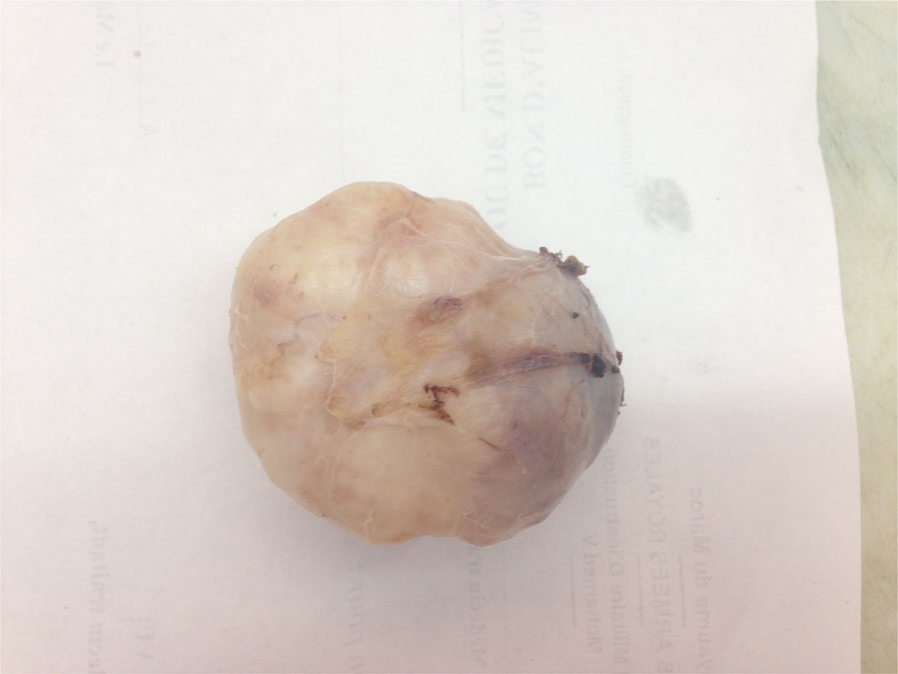
Gross picture of the excised mass

The patient underwent a complete surgical excision of the lesion within its smooth pseudocapsule and without fragmentation (Fig. 3).

**Fig. 4:**
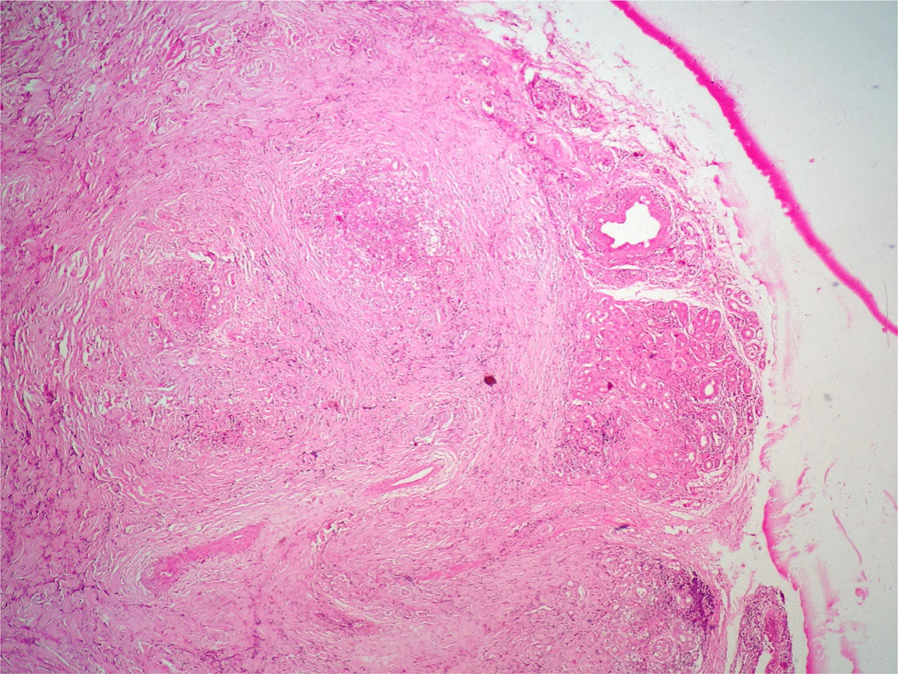
Low magnification showing a lesion with fascicular architecure in a predominantly fibrous background (haematoxylin & eosin stain, × 50)

**Fig. 5:**
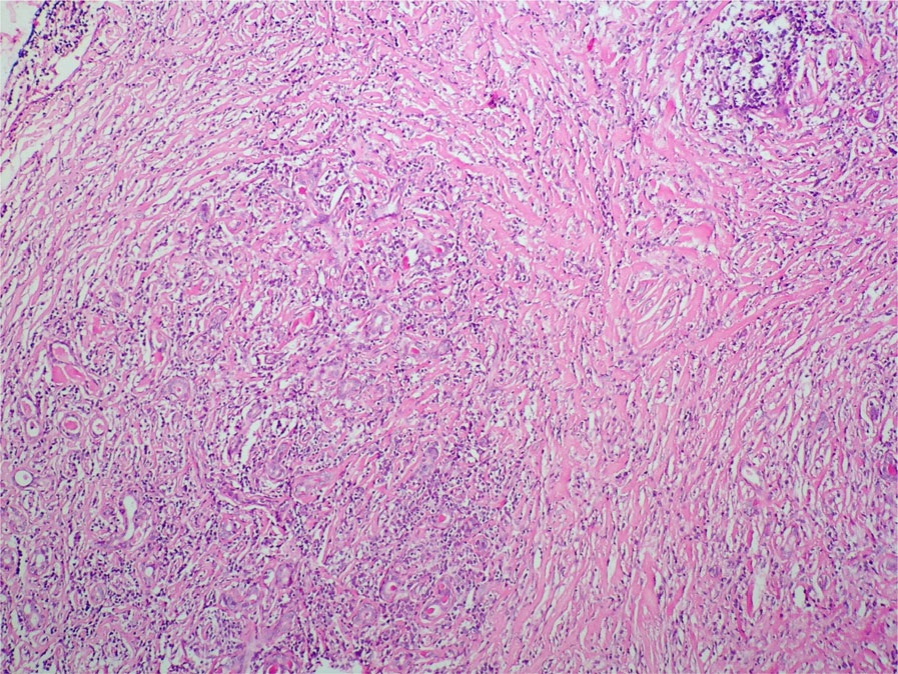
Higher magnification showing the residual glands that are encircled by the proliferation (haematoxylin & eosin stain, × 200)

**Fig. 6:**
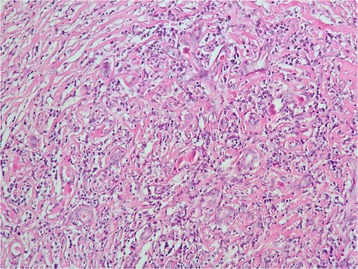
Higher magnification showing a stroma infiltrated by numerous inflammatory cells composed of lymphocytes, plasma cells and eosinophils (haematoxylin & eosin stain, ×400)

Histopathology of the excised mass showed a tumor composed of haphazardly arranged spindle-shaped and oval cells in a predominantly fibrous background, interspersed with prominent dense bands of collagen that clamped residual glands (Figs. 4 and 5). Mitosis and necrosis were absent. The stroma was infiltrated by numerous inflammatory cells composed of lymphocytes, plasma cells and rare eosinophils (Fig. 6). These histopathological features were suggestive of IMT.

**Fig. 7:**
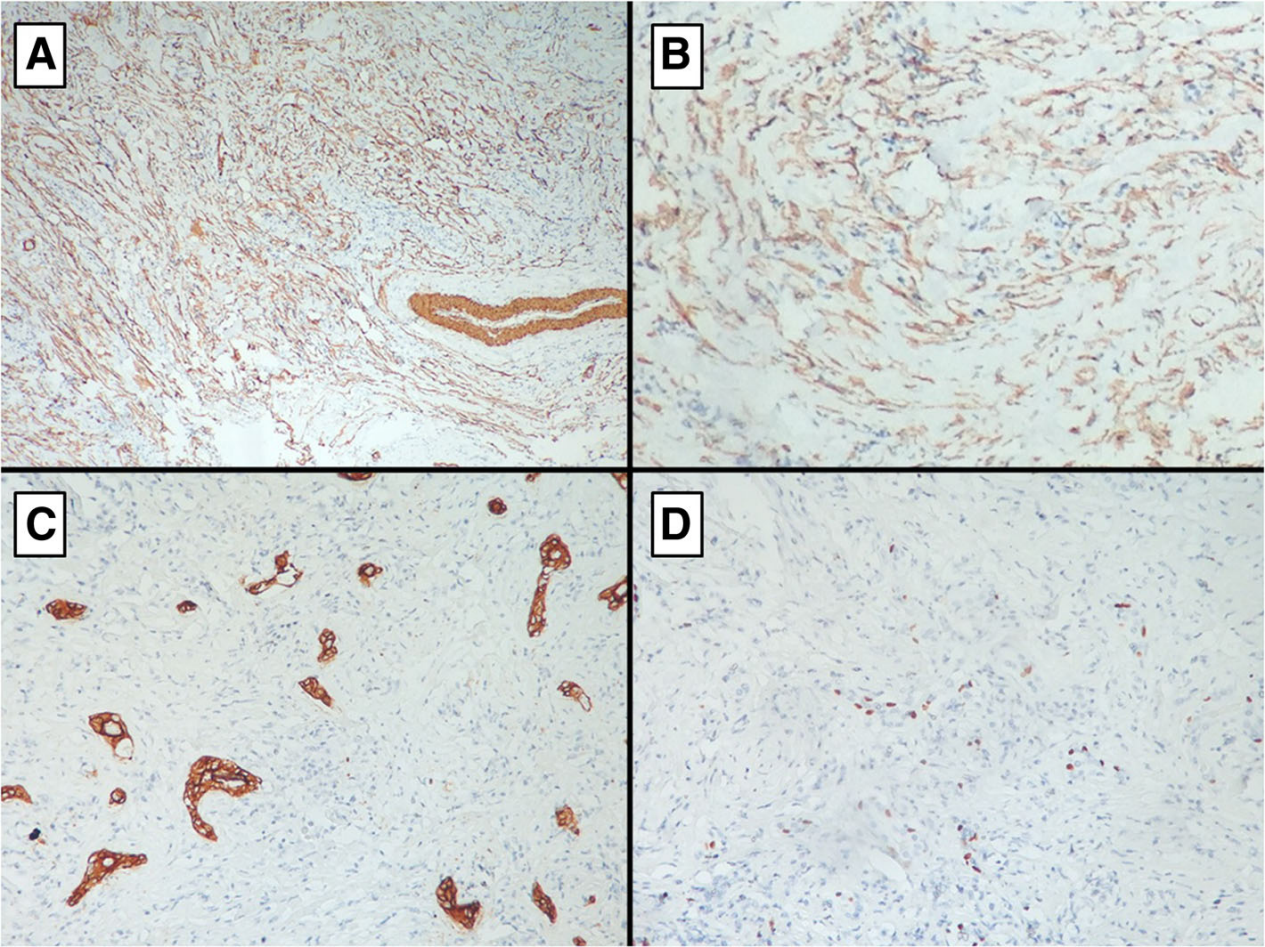
Immunohistochemical staining revealed the expression of smooth muscle actin (**a**) and ALK (**b**) by the neoplastic cells. Keratin highlights the residual glands (**c**). Ki-67 immunostaining showed a low proliferation index (**d**)

The immunohistochemical analysis showed that the spindle-shaped cells to be diffusely positive for smooth muscle actin, vimentin and ALK with a low proliferative index (Ki-67 = 10%) (Fig. 7).

These cells were negative for pancytokeratin AE1/AE3 (positive in residual glands), S100 protein, desmin, CD34.

Co-relating histopathological aspect and immunophenotype profile, a final diagnosis of inflammatory myofibroblastic tumor (IMT) of the lacrimal gland was established.

The patient is currently under regular follow-up with no evidence of recurrence till date (followed for approximately 7 months)

## Discussion

Inflammatory myofibroblastic tumor (IMT) is defined as a benign tumor composed of myofibroblastic spindle cells over an inflammatory background. This lesion has long been reported under many synonyms, including plasma cell granuloma, plasma cell pseudotumour, inflammatory myofibrohistiocytic proliferation, myxoid hamartoma and inflammatory pseudotumour.

This rare neoplasm affects predominantly children and young adults but can also be seen in elderly, with no sex and race predilection [1–3].

IMT occurs most commonly in the lungs, mesentery, pelvis, retroperitoneum, and abdominal soft tissue. Its occurrence in the lacrimal gland is very exceptional [1, 2, 4].

This tumor has historically been reported by many synonyms; such as inflammatory pseudotumor, xanthogranuloma, plasma cell granuloma and plasma cell pseudotumor. This confusion in nomenclature was because of confusion concerning its pathogenesis that whether it is a reactive process or a true neoplasm. Inflammatory reactions to surgery, trauma, irritation and infection are some of triggers for its development. In the 2013 WHO classification, IMTs are recognized as a clonal neoplasm that are considered to have intermediate malignancy that rarely metastasizes. They are characterized by rearrangements involving the ALK (anaplastic lymphoma kinase) gene locus on 2p23 in 50% of cases, leading to constitutive activation of the tyrosine kinase [3, 5, 6].

The mode of presentation of IMT varies. They usually present as a local swelling. Although, systemic symptoms, such as fever, malaise, weight loss and night sweats, are also observed [2, 7].

On computed tomography imaging, IMTs appear as a mildly enhancing soft tissue mass. In general, the radiological features of IMT are variable and non-specific [2, 8].

Grossly, the tumor is well-circumscribed, lobulated or multinodular and may be firm or fleshy with a tan-white cut surface [3, 9, 10].

Characteristic histological findings include the presence of stellate/spindle shaped myofibroblasts and inflammatory spindle cells. The spindled to stellate cells are ranged haphazardly in short or storiform fascicles and exhibit plump, oval nuclei, with no appreciable atypia or hyperchromasia. Cytoplasm is generally sparse or pale eosinophilic in these cells.

Mitotic activity is often low, and atypical mitotic forms are very rare. The stroma shows prominent vasculature and admixed inflammatory cells, consisting chiefly of lymphocytes, plasma cells and occasional neutrophils and eosinophils. Presence of foamy histiocytes has also been reported. Several histological patterns have been described in IMT: loose or myxoid stroma with prominent vascularity; compact spindle cells and densely collagenous with fewer spindle cells and inflammatory cells [1, 10–12]. Histology of our case showed the pattern of densely collagenous stroma with compact spindle cells.

Immunohistochemical study is generally realized to affirm and support the myofibroblastic phenotype of the tumor spindle cells, which consistently express vimentin (99%) and smooth muscle actin (92%). Desmin and other myogenic markers are less frequently expressed and may be focal. Cytokeratins have been identified in about one-third of cases. IMTs are typically negative to myoglobin and S-100 protein [1, 10, 12–14].

In our case also the tumour was strongly positive for vimentin and smooth muscle actin. It was negative for desmin and cytokeratin.

In IMTs, ALK overexpression is typically detectable by immunohistochemistry, which demonstrates cytoplasmic reactivity and is also detectable by FISH [5, 15]. In our case, indeed, tumor cells exhibited cytoplasmic staining with ALK.

The differential diagnosis of IMTs is extensive and broad, comprising nodular fasciitis, smooth muscle and myofibroblastic sarcomas, idiopathic orbitaliInflammation (IOI), inflammatory fibrosarcoma, pseudotumor resulting from mycobacterial infection, gastrointestinal stromal tumours (GIST), fibrous histiocytoma and fibromatosis amongst others [7]. The ALK status has a role in a number of this differential diagnoses. In fact, the majority of this various neoplasms are ALK-negative [16].

Surgery is the mainstay of treatment for IMTs and complete surgical resection, when feasible, is typically curative [2, 11, 12, 17, 18]. Although local Recurrence or progression is site dependent, seen in 25% of extra-pulmonary locations [18, 19]. Several cases have been treated with chemotherapy, Radiotherapy, and/or corticosteroids. Systemic or malignant transformation of IMT is rare and has only been reported in a few cases [2, 11, 17–22].

The recognition of the molecular basis of IMT has led to the increasing use of new biological therapies using inhibitors of the kinase domain of ALK protein with spectacular success and impressive response rate, especially in patients with ALK-positive IMTs [15, 16, 18, 20–22].

## Conclusion

Inflammatory myofibroblastic tumour is a very rare spindle cell neoplasm of intermediate biological potential that may arise in a wide range of anatomic sites. Lacrimal gland represents a distinct and an exceptional location. Differential diagnoses of IMT are varied and immunohistochemistry can play a central role in resolving diagnostic issues. Complete surgical resection is the gold standard.

## Abbreviations

ALK: Anaplastic lymphoma kinase
CT: Computerized tomography
IMT: Inflammatory myofibroblastic tumour
